# Relationships among emotional intelligence, teacher professional identity, and critical thinking disposition in Chinese pre-service teachers

**DOI:** 10.3389/fpsyg.2026.1881577

**Published:** 2026-07-16

**Authors:** Yangyintao Zhao, Azni Yati Kamaruddin, Che Aleha Ladin, Liangrui Ai

**Affiliations:** 1 Faculty of Education, Universiti Malaya, Kuala Lumpur, Malaysia; 2 Faculty of Humanities, Chongqing Metropolitan College of Science and Technology, Chongqing, China

**Keywords:** critical thinking, critical thinking disposition, emotional intelligence, pre-service teachers, teacher professional identity

## Abstract

**Introduction:**

Critical thinking disposition (CTD) reflects a motivational-cognitive tendency to approach complex problems with openness, reflective scepticism, and sensitivity to evidence. Although emotional intelligence (EI) and teacher professional identity (TPI) may be relevant to CTD, their direct and indirect associations, and whether they represent necessary rather than merely predictive conditions for higher CTD, remain unclear.

**Methods:**

A cross-sectional survey was conducted with 1,283 pre-service teachers from three universities in southwest China using validated self-report measures of EI, TPI, and CTD. Partial Least Squares Structural Equation Modelling (PLS-SEM) examined direct and indirect associations, while Necessary Condition Analysis (NCA) assessed whether EI and TPI may represent necessary conditions for higher CTD scores.

**Results:**

EI was positively associated with CTD (*β* = 0.259, *p* < 0.001) and TPI (*β* = 0.466, *p* < 0.001), while TPI was positively associated with CTD (*β* = 0.391, *p* < 0.001). The indirect association between EI and CTD through TPI was statistically significant [*β* = 0.182, 95% CI (0.154, 0.213)]. NCA results further indicated significant moderate necessity effects for EI (*d* = 0.124, *p* < 0.001) and TPI (*d* = 0.147, *p* < 0.001), suggesting that low levels of either condition may constrain higher CTD within the current sample.

**Discussion:**

Interpreted through Self-Determination Theory and viewed from both predictive and necessity-based perspectives, the findings clarify the direct, indirect, and necessity-based associations of EI and TPI with CTD among Chinese pre-service teachers. Efforts to support the development of CTD among pre-service teachers may therefore benefit from combining emotional competence development with opportunities to strengthen professional identity. However, these findings should be interpreted cautiously given the cross-sectional design and the culturally specific single-country sample.

## 1 Introduction

Critical thinking disposition (CTD), the motivational component of critical thinking (CT), reflects a relatively stable willingness to approach complex information with truth-seeking, open-mindedness, reflective scepticism, and sensitivity to evidence (Facione, 1990; Zhao et al., 2024). Whereas CT concerns individuals’ capacity to evaluate information and make reasoned judgements, CTD concerns whether they are inclined to apply that capacity consistently, particularly under uncertainty or when confronted with competing viewpoints (Yurt, 2025; Zhao et al., 2025b). This distinction is especially salient for pre-service teachers, who are still forming the cognitive orientations and professional beliefs that may guide their future judgement and behaviour (Yuan, 2023; Zhao and Ai, 2026). A stronger CTD may make them more willing to question assumptions, tolerate ambiguity, reconsider initial conclusions, and explore alternative interpretations of complex educational problems (Yurt, 2025; Zhao et al., 2025a). CTD, therefore, represents an important psychological foundation for reflective and intellectually responsible thinking among pre-service teachers. Williams (2010) also emphasised that democratic education aimed at fostering civic morality should prioritise cultivating pre-service teachers’ CT attitudes and attentiveness, thereby ensuring a reservoir of high-calibre intellectual talent for society at large.

Against this backdrop, it is important to identify the psychological resources that may be associated with CTD among pre-service teachers. Emotional intelligence (EI) and teacher professional identity (TPI) warrant particular attention because they reflect two complementary psychological processes: the regulation of emotional experiences and the incorporation of a professional role into the self-concept. EI refers to individuals’ perceived capacity to recognise, understand, use, and regulate their own and others’ emotions (Salovey and Mayer, 1990; Yin et al., 2013). For pre-service teachers, this capacity may be particularly relevant during periods of role transition and emotionally demanding social interaction, as it may help them regulate affective responses and maintain reasoned judgement. TPI, by contrast, refers to the extent to which individuals identify with the teacher role and internalise its associated values, responsibilities, beliefs, and commitments (Ding et al., 2022; Beijaard et al., 2023). As a developing form of role identity, TPI may influence how pre-service teachers understand themselves, derive meaning from their anticipated professional role, and regulate their motivation and behaviour. Although conceptually distinct, EI and TPI may therefore provide complementary affective and identity-related psychological foundations for reflective, open-minded, and evidence-sensitive thinking.

Despite their potential relevance, the relationships of EI and TPI with CTD remain insufficiently examined among pre-service teachers. EI may help individuals maintain emotional stability, cognitive openness, and reasoned judgement when confronted with stress, interpersonal conflict, or competing information (Yin et al., 2013; Yin, 2015; Özdemir Cihan and Dilekmen, 2024; Zhao and Ai, 2026). TPI may provide motivational coherence and a stable sense of self-relevance, thereby supporting sustained engagement, responsibility, and reflection when individuals encounter role-related challenges (Ding et al., 2022; Rosenfeld and Yemini, 2023; Zeng and Liu, 2024). Existing evidence further suggests that stronger EI may be associated with a more positive professional identity through more adaptive emotional experiences and favourable evaluations of one’s professional role (Butakor et al., 2021; Su et al., 2024). EI and TPI may therefore be associated with CTD through both direct and indirect statistical pathways, with TPI serving as an indirect explanatory link between EI and CTD.

Prior research has begun to clarify these relationships. Zhao et al. (2025a) reported a positive association between EI and CTD among Chinese pre-service teachers, with resilience serving as a possible mediator. However, resilience primarily represents an adaptation-related psychological resource, whereas TPI reflects the internalisation of professional values and role commitments. It therefore remains unclear whether an identity-based pathway through TPI may also help explain the EI-CTD association. This question is particularly relevant to pre-service teachers, whose professional identities are still developing as they transition towards the teaching profession (Beijaard et al., 2023; Zeng and Liu, 2024). Moreover, prior research has largely remained within predictive frameworks, leaving unexamined whether EI or TPI may function as necessary conditions for attaining higher CTD.

A necessity-based perspective is theoretically meaningful for understanding CTD because it examines whether attaining higher levels of this disposition depends on minimum psychological conditions. Conventional predictive models generally treat psychological resources as additive contributors, assuming that higher EI or TPI is associated with higher CTD and that weakness in one resource may be compensated for by other favourable factors (Dul, 2016). However, CTD reflects a sustained willingness to engage in reflective and open-minded thinking, which may depend on certain foundational psychological conditions. EI may constitute an affective foundation for maintaining critical engagement when emotions interfere with information processing (Zhao and Ai, 2026), while TPI may constitute an identity-based foundation through which reflective and evidence-sensitive thinking becomes internalised as part of the emerging teacher role (Zeng and Liu, 2024). If these resources are necessary, their theoretical role is asymmetrical: sufficient EI or TPI may not guarantee higher CTD, but insufficient levels would place an upper limit on the CTD that can be attained (Richter et al., 2023). Examining necessity, therefore, extends the understanding of EI and TPI from beneficial correlates of CTD to potential boundary conditions on the attainment of higher CTD. Necessary condition analysis (NCA) was used to test this possibility and to identify sample-specific bottlenecks that conventional predictive analysis cannot reveal (Dul, 2016; Richter et al., 2023).

Accordingly, this study extends the traditional correlational analysis perspective by incorporating a necessity-based approach, providing a more comprehensive and in-depth examination of the relationships and necessary linkages among EI, TPI, and CTD within pre-service teachers. It aims to provide a more integrated understanding of the underlying interplay among these constructs and to identify essential factors that may inform both theoretical advancement and practical strategies for cultivating pre-service teachers’ CTD. These objectives are addressed through three related questions concerning the associations among the constructs, the indirect role of TPI, and the potential necessity of EI and TPI for higher CTD. The following specific research questions (RQs) were therefore proposed:

*RQ1*: What are the relationships among EI, TPI, and CTD in pre-service teachers?
*RQ2*: Does TPI statistically mediate the association between EI and CTD?
*RQ3*: Do EI and TPI constitute necessary conditions for CTD in the current sample?

## 2 Literature review

### 2.1 Critical thinking disposition

The intellectual roots of CTD can be traced to philosophical traditions that emphasise a movement from passive acceptance towards active questioning. Socratic dialogue, Enlightenment scepticism, and Deweyan pragmatism all highlight the educational value of questioning assumptions, attending to evidence, and maintaining a reflective orientation towards knowledge and social practice (Griffin et al., 2003; Piro et al., 2015; Wang, 2017). Ennis (1987) portrayed CTD as an active endorsement of the norms of rational enquiry, driven by an intrinsic commitment to logic, evidence, and open-mindedness. Facione (1990) further framed CTD as a constellation of thinking habits, such as truth-seeking, systematicity, and prudence, thereby rendering the construct observable and measurable. Later perspectives extended the concept by emphasising ethical respect for reason, justice, and coherence (Siegel, 1999), as well as a disposition to question taken-for-granted social and educational assumptions within critical pedagogy (Brookfield, 2011). Taken together, these philosophical commitments were subsequently conceptualised as the psychological construct of CTD and adopted in teacher education research to examine how future teachers orient themselves towards enquiry, evidence, and professional uncertainty. For the present study, CTD is defined as a relatively stable motivational-cognitive disposition characterised by critical openness and reflective scepticism, including a willingness to consider alternative perspectives, question claims, and re-evaluate one’s own reasoning (Sosu, 2013).

As a foundational psychological orientation underpinning reflective and evidence-sensitive professional learning, CTD is particularly relevant among pre-service teachers (Yang, 2012; Yuan, 2023). It reflects pre-service teachers’ tendency to maintain intellectual openness, question taken-for-granted assumptions, consider alternative viewpoints, and remain attentive to evidence in complex educational contexts (Yang, 2012; Leibovitch et al., 2025; Zhao et al., 2025a). CTD may also orient pre-service teachers towards greater epistemic responsibility by encouraging them to examine their own beliefs and assumptions rather than relying solely on habitual or authority-based judgements (Uluçınar and Aypay, 2018; Moore, 2019). Such demands commonly arise during coursework and practicum, when pre-service teachers must reconcile competing pedagogical perspectives, interpret ambiguous student responses, address diverse learning needs, or revise instructional plans following feedback (Zhao and Ai, 2026). These situations exemplify the “wicked problems” of teaching because they involve incomplete information, competing considerations, and no single correct solution (Jain et al., 2024). In such contexts, CTD reflects a willingness to remain engaged in reasoned deliberation even when available evidence does not support a straightforward resolution.

Building on these conceptual foundations, recent empirical research has examined CTD within contemporary teacher education contexts. Among pre-service teachers, studies have primarily concentrated on the associations between CTD and various psychological or cognitive attributes such as self-efficacy, flexibility, and mindfulness (Aydin Gürler, 2021; Chen et al., 2024; Gökçe and Güner, 2024; Yurt, 2025), while a growing body of studies has also begun to explore the potential of pedagogical interventions, such as sustainable multicultural environment teaching and digital narrative reflection methods, to enhance CTD for pre-service teachers (Belda-Medina, 2022; Sutoyo et al., 2023). Diverse teaching structures and classroom atmospheres with varying backgrounds have also been found to support pre-service teachers’ willingness to engage in autonomous and evidence-oriented thinking (Jintalan and Litao, 2024). Despite these contributions, limited attention has been paid to the associations of EI and TPI with CTD among pre-service teachers. The interrelationships among these constructs also remain insufficiently understood in the context of the emotional and professional demands of teaching. Furthermore, whether EI and TPI may constitute necessary conditions for higher CTD remains unexplored.

### 2.2 Emotional intelligence

EI denotes the integrated capacity to perceive and interpret emotional cues accurately, regulate their intensity and direction flexibly, and deploy emotional resources to support thoughts, motivation, and social interaction (Salovey and Mayer, 1990; Su et al., 2024). Existing scholarship distinguishes ability, trait, and mixed models of EI. Ability models conceptualise EI as a set of emotion-processing abilities commonly assessed through performance-based tasks, whereas trait models emphasise individuals’ self-perceived emotional dispositions; mixed models combine emotional abilities with broader motivational and social competencies (Yin et al., 2013). As the present large-sample cross-sectional survey focuses on pre-service teachers’ perceptions of their typical emotional functioning, EI was operationalised using the self-report Wong and Law Emotional Intelligence Scale (WLEIS; Law et al., 2004). The instrument assesses perceived capacities to appraise one’s own and others’ emotions, use emotions, and regulate one’s own emotions. This approach captures perceived typical functioning rather than maximal performance on standardised emotion-processing tasks and is consistent with several established self-report applications involving in-service and pre-service teachers (Yin et al., 2013; Turner and Stough, 2020; Chen and Xie, 2025; Zhao et al., 2025a).

EI is particularly relevant to the emotional and interpersonal demands of teaching. Teachers have to manage both their private affective states and their public emotional displays across classroom instruction, parent-school communication, and collegial collaboration (Yin et al., 2013; Yin, 2015). Those with stronger EI may be more capable of engaging in deep acting by genuinely generating emotions aligned with pedagogical goals, thereby reducing emotional dissonance (Su et al., 2024). Emotional awareness and empathy may also help teachers detect students’ subtle affective shifts, use supportive verbal and non-verbal behaviours, and respond constructively in conflictual or culturally diverse situations, contributing to a more stable classroom climate and stronger classroom-management confidence (Jennings and Greenberg, 2009; Valente et al., 2022; Li et al., 2024).

EI is also relevant to teachers’ professional well-being and reflective functioning. Greater emotional regulation has been associated with lower burnout and turnover intentions, as well as higher teaching satisfaction and professional well-being (Mérida-López and Extremera, 2017; Li et al., 2024). Among pre-service teachers, these capacities may be especially important during practicum and role transition, when unexpected emotional incidents can disrupt psychological balance and instructional focus (Vesely et al., 2014; Turner and Stough, 2020). Sensitivity to emotional cues may further enable them to use affective information as feedback for refining pedagogical approaches and future instructional responses (Hussain Garma, 2024).

### 2.3 Teacher professional identity

TPI concerns how individuals understand themselves in relation to the teaching profession and internalise its values, responsibilities, and expectations (Beijaard et al., 2023; Li and Khairani, 2025). From a personal identity perspective, it develops through the interpretation of prior experiences, beliefs, motivations, and perceptions of professional competence. From a social identity perspective, it is also shaped through interaction with mentors, peers, institutions, and broader societal expectations surrounding the teacher role (Rushton and Reiss, 2021; Li and Khairani, 2025). TPI is therefore not a fixed attribute but an ongoing process of negotiation, in which individuals continually interpret and revise their professional self-understandings in response to experience, feedback, social recognition, and changing role expectations. For the present study, TPI is operationalised as pre-service teachers’ self-reported identification with the teaching profession, reflected in their professional self-efficacy, knowledge about the profession, and professional commitment (Nickel and Crosby, 2022; Zeng and Liu, 2024).

For pre-service teachers, this ongoing identity negotiation is especially salient during academic learning and practicum, as they begin to interpret the meaning, value, and responsibilities of the teaching role (Beijaard et al., 2023). TPI is not merely a form of professional self-awareness, but rather constitutes an essential psychological preparation and developmental foundation for pre-service teachers entering the education profession (Long et al., 2026). A strong sense of professional identity can enhance pre-service teachers’ learning motivation, enabling them to remain resilient and committed when confronted with complex educational tasks and challenges (Hong, 2010). In addition, TPI facilitates pre-service teachers’ integration into the educational community, encouraging active participation in collaboration and exchange, and enabling them to accumulate social capital and professional resources (Beijaard et al., 2004). Through these motivational and professional resources, a stronger TPI may be associated with greater willingness to engage in reflective enquiry, consider alternative viewpoints, and evaluate pedagogical judgements against evidence, all of which are dispositional tendencies closely aligned with CTD.

### 2.4 Emotional intelligence and critical thinking disposition

The emotion-regulation component of EI may be particularly relevant to CTD because it can limit the extent to which anxiety, frustration, or defensiveness disrupts attention and contributes to premature judgements. When affective reactions are managed more effectively, individuals may be better able to pause before responding, compare competing interpretations, and reconsider conclusions that are inconsistent with available information (Salovey and Mayer, 1990; Christodoulakis et al., 2023). These processes support cognitive flexibility and openness to revision, which are central to CTD (Facione, 1990; Zhao et al., 2025a).

Cross-sectional studies by Kang (2015), Sk and Halder (2020), and Christodoulakis et al. (2023) consistently reported positive associations between EI and CTD among university students. Taken together, their samples included students at both undergraduate and postgraduate levels, broadening the range of educational levels represented in the existing evidence. Longitudinal evidence has further substantiated this hypothesis, revealing that specific fundamental components of EI have enduring and far-reaching effects on CTD (Kaya et al., 2018). Nevertheless, existing evidence predominantly derives from student populations in healthcare or multidisciplinary domains, with empirical investigations focusing specifically on pre-service teachers remaining notably scarce. Moreover, most of these studies have focused primarily on the direct relationship between EI and CTD, with limited exploration of potential indirect pathways. In addition, they have largely remained at the correlational level, while the research into whether EI may constitute a necessary condition for CTD has yet to be adequately explored. Accordingly, the study proposes the following research hypothesis:

*H_1_*: Pre-service teachers’ EI is positively and significantly associated with their CTD.

### 2.5 Emotional intelligence and teacher professional identity

EI is likely to relate positively to TPI because teachers’ professional identity is closely influenced by emotional experiences, interpersonal interactions, and perceived professional competence (Su et al., 2024). Teachers with high EI are more likely to regulate their emotions proactively and maintain a positive state of mind (Zhao et al., 2025a). Higher EI also enhances individuals’ monitoring of their emotional states, enabling teachers to face challenges with greater confidence and a heightened sense of professional competence, an attribute that facilitates identification with the teaching role (Palomera et al., 2008; Rodrigues and Mogarro, 2019). Moreover, TPI is generally co-constructed through social interaction and emotional experience (Su et al., 2024), and EI supplies the emotional regulation and interpersonal adaptability that enable pre-service teachers to gain identity affirmation and a sense of recognition within diverse encounters. Prior studies have suggested that TPI is closely associated with teachers’ emotional experiences (Su, 2025), that daily affective encounters may influence identity formation (Rodrigues and Mogarro, 2019), and that teachers possessing stronger EI may be more capable of recognising and managing such emotional experiences. A large-scale survey of Chinese secondary-school teachers found that EI significantly predicted professional identity (Su et al., 2024), while evidence from Ghana indicated that EI showed both direct and indirect links with TPI through job satisfaction (Butakor et al., 2021). Recent qualitative evidence from Chinese university English teachers further suggests that the relationship between emotional functioning and professional identity may depend on contextual conditions, including workload, institutional support, career-development opportunities, social recognition, and student feedback (Yao et al., 2025). Longitudinal evidence further indicates that increases in teachers’ positive affect are associated with stronger professional identity, greater teaching enthusiasm, and enhanced professional commitment (Long et al., 2026). Accordingly, the following hypothesis is formulated:

*H_2_*: Pre-service teachers’ EI is positively and significantly associated with their TPI.

### 2.6 Teacher professional identity and critical thinking disposition

TPI may also be positively associated with CTD. TPI reflects the extent to which individuals come to embrace the professional values, role responsibilities, and expectations associated with teaching (Ding et al., 2022). Unlike broader personal or academic identities, TPI is specifically organised around the norms, responsibilities, and judgement demands of teaching. It is therefore particularly relevant to CTD because it may shape whether pre-service teachers regard evidence evaluation, reflective enquiry, and the revision of pedagogical judgements as integral to being a teacher (Facione, 1990; Beijaard et al., 2004). In a positive teaching environment, pre-service teachers who develop a strong sense of identification with teaching are likely to show greater willingness to endorse professional values for reasoned pedagogical judgement (Zhao et al., 2025a). In a reflection-oriented professional context, professional identity may contribute to task value and persistence, thereby strengthening pre-service teachers’ willingness to participate in peer dialogue, reconsider initial assumptions, and remain open to different interpretations of educational problems. These dispositional tendencies are closely aligned with the core qualities of CTD (Facione, 1990). Furthermore, a well-developed professional identity has frequently been linked to professional self-efficacy (Hong, 2010), which may support a more confident and active orientation towards engaging in critical and reflective thinking when facing complex pedagogical problems (Zhao et al., 2024).

Empirical studies provide preliminary, albeit indirect, support for this association. In a Chinese EFL sample, TPI was found to be significantly related to teachers’ CT and career success (Ding et al., 2022). Studies in Iranian EFL contexts have also reported associations between TPI, CT skills, and critical pedagogy (Moslemi and Habibi, 2019; Zarei and Dobakhti, 2023). Although these studies did not measure CTD directly, they remain relevant because CTD represents the motivational basis for applying CT skills and engaging with critical pedagogy (Facione, 1990; Zhao et al., 2024). Their findings therefore provide indirect evidence that TPI may also be associated with CTD. Su et al. (2024) further noted that educators with a firmly developed professional identity are more inclined to commit to ongoing professional learning, respond adaptively to curricular change, and maintain a reflective orientation within their teaching practice. In light of the reviewed literature, the following hypothesis is advanced:

*H_3_*: Pre-service teachers’ TPI is positively and significantly associated with their CTD.

### 2.7 Teacher professional identity as a potential mediator

Self-Determination Theory (SDT) may provide an integrative explanation for why TPI could mediate the EI-CTD relationship in this group. SDT posits that when the three basic needs for autonomy, competence, and relatedness are fulfilled, individuals tend to develop stronger intrinsic motivation and to internalise external regulations and roles, thereby exhibiting more adaptive psychological functioning and positive behavioural outcomes (Deci et al., 2017). Empirical applications of SDT in teacher-learning contexts have also examined basic psychological need satisfaction during student teachers’ initial teaching experiences and the role of autonomous motivation in teachers’ engagement in professional learning (Evelein et al., 2008; In de Wal et al., 2014). EI can be viewed as a personal emotional resource that may support need-satisfying experiences among pre-service teachers. Individuals with stronger EI may regulate adverse emotions more effectively during role adjustment, which may support their sense of autonomy (Costa et al., 2018). Effective emotional interactions with students and mentors during teaching practice, together with positive feedback, may be associated with an enhanced sense of competence (Zhao et al., 2025a). Moreover, EI may also be related to stronger interpersonal sensitivity, enabling pre-service teachers to build meaningful relationships with colleagues and supervisors, which may contribute to their need for relatedness (Schutte et al., 2001). Once these needs are sufficiently met, pre-service teachers may be more inclined to internalise the professional significance of the teaching role and develop a more consolidated professional identity. An internalised professional identity may further facilitate a shift in pre-service teachers’ professional learning motivation from externally driven task completion towards a more intrinsically motivated pursuit of professional growth (Deci et al., 2017). This intrinsic motivation may be linked to a greater willingness to engage in reflective teaching practice, question existing assumptions, and consider improved instructional approaches. Such dispositional orientations represent core characteristics of CTD (Facione, 1990; Zhao and Ai, 2026). In this sense, TPI may serve as an indirect explanatory link through which EI is associated with CTD. Accordingly, the following hypothesis is proposed.

*H_4_*: Pre-service teachers’ TPI mediates the association between their EI and CTD.

**Figure 1:**
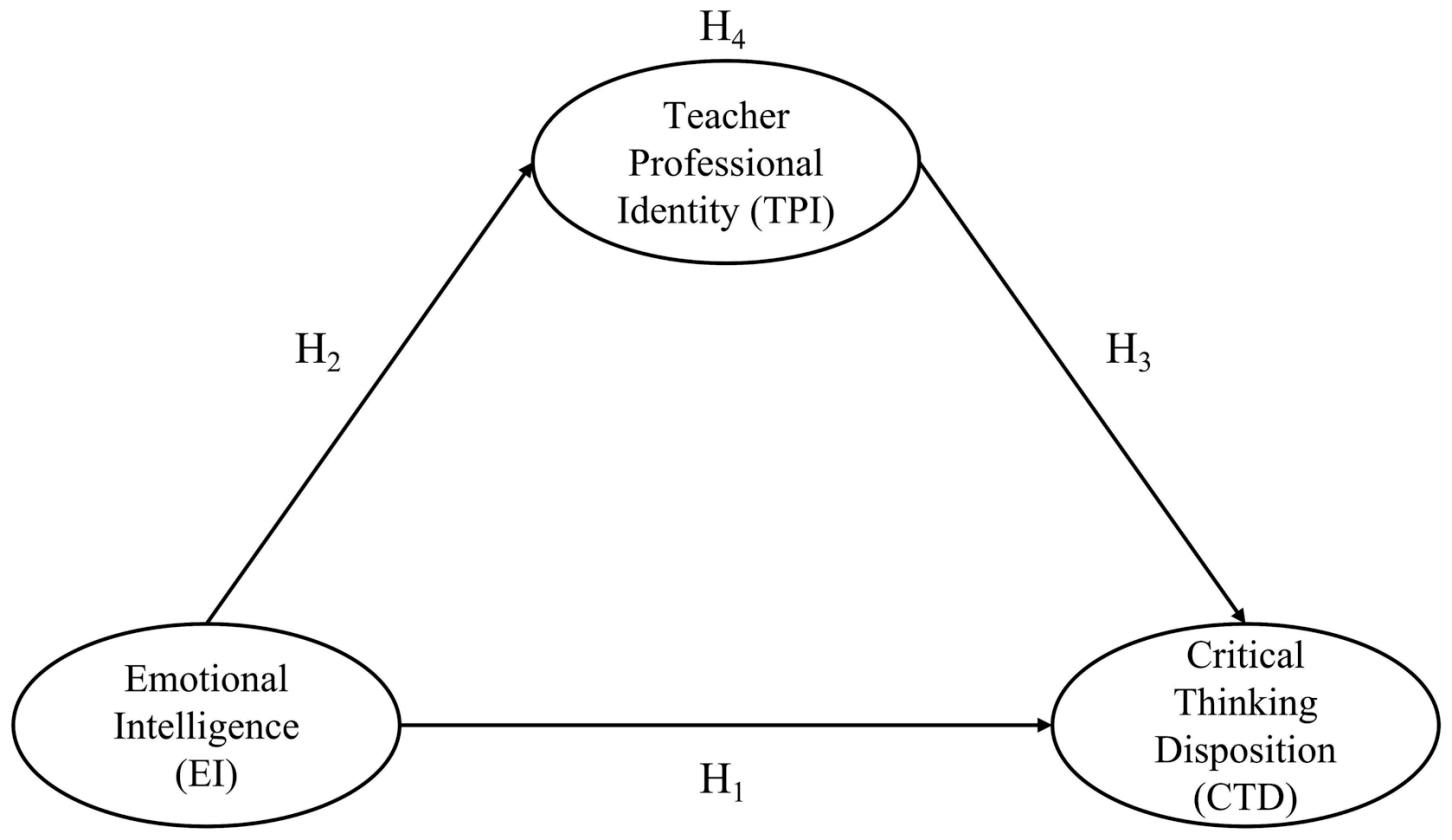
Proposed conceptual model.

Figure 1 presents the proposed conceptual model and summarises the hypothesised relationships examined in the present study.

## 3 Methodology

### 3.1 Participants

The study was conducted in south-western China, where educational development remains comparatively uneven and strengthening regional educational capacity continues to receive policy attention. Using cluster sampling, 1,336 pre-service teachers were recruited from three universities that offered established teacher training programmes. The pre-service teachers were either currently undertaking or had already completed systematic training in educational knowledge. All participants completed the online questionnaire voluntarily, following established ethical standards and confidentiality guidelines. Before data collection, formal approval for ethical compliance was secured. The researchers then reviewed all responses after data collection to ensure data quality. Responses were excluded if they selected the same response option for all substantive questionnaire items, followed a clearly repeated response sequence across the scales, or completed the questionnaire in less than 5 min, approximately one-third of the expected completion time. After excluding 53 invalid responses, 1,283 valid cases (Mage = 20.58, SD = 1.73) were retained for the final analysis. Table 1 summarises the final sample, which included participants across different genders, ages, academic years, and major categories. The majority of participants fell within the 18–23 age range, and the distribution across academic years was relatively balanced. In terms of disciplinary composition, the sample included pre-service teachers from Pedagogy, Early Childhood Education, Mathematics Education, and Music Education.

**Table 1:** Overview of sample characteristics.

Respondent profile	Classification	*N*	Percentage (%)
Gender	Male	379	29.5
	Female	904	70.5
Age	18–20	621	48.4
	21–23	639	49.8
	24–25	23	1.8
Grade	1st year	343	26.7
	2nd year	312	24.4
	3rd year	303	23.6
	4th year	325	25.3
Major	Pedagogy	298	23.2
	Early Childhood Education	428	33.4
	Mathematics Education	253	19.7
	Music Education	304	23.7

N, Number.

### 3.2 Measures

Data were gathered via an online questionnaire as part of a cross-sectional design. It includes demographic items and self-report scales for EI, TPI, and CTD. Participants accessed the survey by scanning a QR code, and the entire completion process took roughly 12–15 min. The English-language instruments were adapted into Chinese using the procedure outlined by Brislin (1970). One bilingual researcher prepared the Chinese versions, while another independently rendered them back into English. The two English versions were subsequently compared, and any inconsistencies were resolved through discussion. The resulting Chinese items were then evaluated by an education specialist, with minor wording adjustments made to enhance comprehensibility and contextual suitability. Detailed information on each measure is provided below.

#### 3.2.1 Emotional intelligence

EI was measured using the 16-item Wong and Law Emotional Intelligence Scale (WLEIS; Law et al., 2004). The scale utilises a seven-point Likert format, with responses ranging from strong disagreement to strong agreement. It consists of four subdimensions: self-emotion appraisal (SE), others-emotion appraisal (OE), use of emotion (UE), and regulation of emotion (RE), with four items assigned to each subdimension. Example items include statements such as ‘I am a self-motivating person’ and ‘I am quite capable of controlling my own emotions’. The overall Cronbach’s alpha coefficient for EI was 0.876.

#### 3.2.2 Teacher professional identity

TPI was evaluated using a 13-item Teacher Professional Identity Scale (TPIS) developed by Zeng and Liu (2024) specifically for Chinese pre-service teachers. The instrument employs a five-point Likert scale ranging from strongly disagree to strongly agree. It includes three subdimensions: professional self-efficacy (PS; five items), knowledge about the profession (KP; four items), and professional commitment (PC; four items). Sample items include statements such as ‘I believe I can do well in my teaching career’ and ‘It is important for me to be a member of the teaching profession’. The total scale demonstrated good internal consistency with a Cronbach’s alpha of 0.851.

#### 3.2.3 Critical thinking disposition

CTD was assessed with Sosu’s (2013) 11-item Critical Thinking Disposition Scale (CTDS). The CTDS was selected because its dimensions of critical openness and reflective scepticism closely correspond to the conceptualisation of CTD adopted in this study. Its concise 11-item format also limited respondent burden within a questionnaire assessing several psychological constructs. Although broader instruments encompass additional facets, the CTDS provided a focused and parsimonious measure aligned with the present research aims. Responses were recorded on a five-point Likert scale ranging from strongly disagree to strongly agree. The instrument comprises two subdimensions: critical openness (CO; seven items) and reflective scepticism (RS; four items). Sample items include statements such as ‘I am often on the lookout for new ideas’ and ‘I often re-evaluate my experiences so that I can learn from them’. The overall Cronbach’s alpha was 0.850.

### 3.3 Common method variance test

Several procedural measures were implemented during data collection to reduce potential common method bias. Participation was voluntary, no personally identifying information was collected, and respondents were assured that their responses would remain confidential and be used solely for research purposes. Then, potential common method variance was preliminarily assessed using Harman’s (1976) single-factor test. The unrotated exploratory factor analysis showed that the first factor accounted for 24.07% of the total variance, below the commonly used threshold of 40%. This finding suggests that no single factor dominated the covariance among the measured variables. However, given the recognised limitations of Harman’s single-factor test, the result was treated only as an initial diagnostic rather than definitive evidence that common method variance was absent.

### 3.4 Analytical methods

Partial Least Squares Structural Equation Modelling (PLS-SEM) and Necessary Condition Analysis (NCA) were used for different but complementary analytical purposes. PLS-SEM was employed to examine whether EI and TPI were positively associated with CTD on average, and whether TPI statistically mediated the association between EI and CTD. In contrast, NCA was used to examine whether insufficient levels of EI and TPI constrained the possibility of achieving higher CTD scores. Thus, PLS-SEM addresses a predictive question, namely whether changes in antecedents are associated with changes in the outcome, whereas NCA addresses a necessity question, namely whether a minimum level of an antecedent is required before a high level of the outcome can be observed (Richter et al., 2023). Using both approaches therefore enables the study to identify factors that are predictive of CTD and factors that may operate as limiting conditions for higher CTD.

#### 3.4.1 Assessing the relationships among EI, TPI, and CTD

PLS-SEM was used to test the proposed links between EI, TPI, and CTD. This methodological choice was deemed suitable given the prediction-oriented nature of the study, focusing on the extent to which EI and TPI explained variance in CTD and whether TPI may serve as an indirect explanatory link. In addition, EI, TPI, and CTD were modelled as higher-order constructs (HOCs) reflected by their respective subdimensions, which served as lower-order constructs (LOCs). The LOCs were specified reflectively at the item level, while the HOCs were also specified reflectively because their subdimensions were treated as manifestations rather than defining components of the corresponding constructs. Thus, all three models were reflective-reflective HOCs (Hair, 2017). PLS-SEM is well-suited to estimating such reflective-reflective higher-order structures, particularly when the analytical focus is on measurement quality, path coefficients, explanatory power, effect sizes, and predictive relevance rather than solely on covariance-based global model fit (Hair, 2017). Moreover, PLS-SEM enabled the extraction of latent variable scores for subsequent NCA, ensuring consistency between the predictive and necessity-based analyses (Richter et al., 2023).

Following Sarstedt et al. (2019), a disjoint two-stage procedure was adopted. At the first stage, all LOC measurement models were examined in terms of reliability and validity. The resulting latent variable scores were subsequently employed as indicators for their respective HOCs. In the second stage, the HOC measurement models were evaluated, followed by structural path analysis. The indirect association through TPI was tested using bootstrapping with 5,000 resamples (Hair, 2017).

#### 3.4.2 Assessing the necessity of EI and TPI

**Figure 2:**
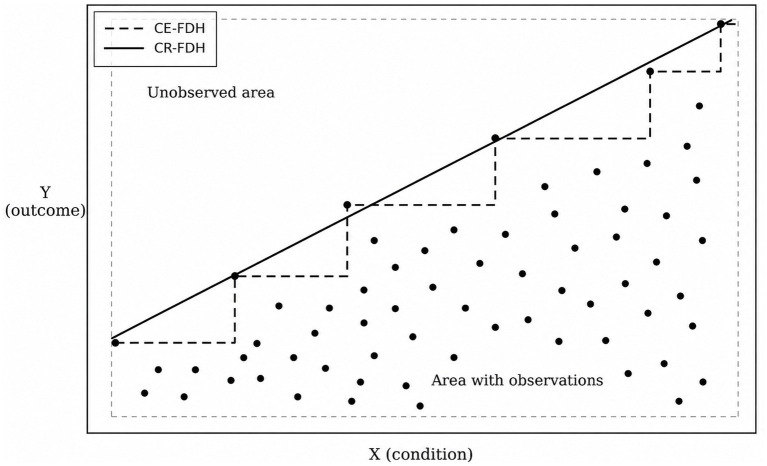
Illustrative scatterplot for NCA [Adapted from Dul et al. (2023)].

This study further employed NCA to examine whether EI and TPI might function as necessary conditions for pre-service teachers’ CTD within the current sample, using the NCA module in SmartPLS 4.0. Following Dul’s (2016) procedure, scatterplots were generated to depict the necessity relationships linking conditions (X) and the outcome (Y), as shown in Figure 2. An evident space in the plot’s upper-left region suggests that high or desirable outcome values seldom occur when condition variables remain at relatively low levels; higher outcome levels are attainable only if the conditions satisfy a minimum threshold, implying potential necessity (Dul, 2016). To verify the boundary of feasible outcomes, a ceiling line was fitted using either the Ceiling Envelopment-Free Disposal Hull (CE-FDH) or the Ceiling Regression-Free Disposal Hull (CR-FDH) method. This study employed CE-FDH, as it offers higher precision and broader applicability across research contexts (Richter et al., 2023). This choice was also consistent with the present data, as the PLS-derived latent variable scores displayed irregular and stepwise upper boundaries, which were more appropriately represented by the non-parametric CE-FDH approach than by CR-FDH. The ceiling boundary separates feasible from theoretically impossible values, identifying the condition thresholds. The necessity effect size (d) was derived by comparing the empty area with the overall empirical space, with values ≥ 0.10 (medium level) and statistical significance (*p* < 0.05, 10,000 resamples) indicating necessity (Dul et al., 2020). Lastly, bottleneck analysis was conducted to identify the minimum EI and TPI values needed for attaining varying CTD thresholds.

## 4 Results

### 4.1 Preliminary analysis

Table 2 reports descriptive statistics and inter-construct correlations for the core latent constructs. The descriptive results show that the mean score for EI was 5.22 (SD = 1.01), while TPI and CTD had corresponding means of 3.46 (SD = 0.78) and 3.71 (SD = 0.82). The correlation results showed significant positive correlations across all key variables (*p* < 0.001). Specifically, EI was positively correlated with TPI (*r* = 0.463) and CTD (*r* = 0.438). Furthermore, TPI and CTD were likewise positively correlated (*r* = 0.511). These results offer initial empirical support for the hypothesised relationships and warrant subsequent structural model analysis.

**Table 2:** Descriptive statistics and inter-construct correlations.

Latent construct	M ± SD	1	2	3
1. EI	5.22 ± 1.01	–		
2. TPI	3.46 ± 0.78	0.463***	–	
3. CTD	3.71 ± 0.82	0.438***	0.511***	–

***p < 0.001, M, Mean; SD, Standard Deviation.

### 4.2 Test of the PLS-SEM measurement model

Initially, the measurement models for all LOCs were assessed (see Table 3). All indicators showed factor loadings above 0.70, and all average variance extracted (AVE) estimates were above 0.50, supporting adequate convergent validity (Hair et al., 2019). Internal consistency was evaluated through Cronbach’s alpha (CA) and composite reliability (CR), which are all greater than 0.70. Discriminant validity results are reported in Table 4. Under the Fornell-Larcker criterion, the AVE square roots, shown along and below the diagonal, exceeded the largest correlations between constructs. The heterotrait-monotrait ratio (HTMT) values, reported above the diagonal, were all below 0.85, supporting robust discriminant validity (Hair et al., 2019). Given these findings, latent variable scores for all LOCs were obtained to represent their respective HOCs. The HOCs’ measurement models were then assessed, with results in Tables 3, 4 confirming acceptable reliability and validity for further structural analysis. Although the HOC-level CA values for CTD (0.608) and TPI (0.692) were slightly below the conventional 0.70 threshold, Cronbach’s alpha is sensitive to the number of indicators and may be underestimated when constructs are represented by only a few LOCs (Cho, 2016). Methodological literature therefore recommends evaluating HOC reliability using multiple criteria rather than relying on alpha alone, with CR generally providing a more suitable estimate in PLS-SEM (Hair, 2017; Sarstedt et al., 2019). Given that the corresponding CR, HOC loadings, and AVE values met the recommended criteria, both HOC measurement models were considered acceptable.

**Table 3:** Summary of reliability and convergent validity.

LOC	Items	Indicator loading	CA	CR	AVE	LOC	Items	Indicator loading	CA	CR	AVE
SE	SE1	0.721***	0.735	0.834	0.556	PS	PS1	0.803***	0.834	0.883	0.601
	SE2	0.753***					PS2	0.742***			
	SE3	0.772***					PS3	0.762***			
	SE4	0.736***					PS4	0.783***			
OE	OE1	0.755***	0.785	0.861	0.607		PS5	0.785***			
	OE2	0.762***				KP	KP1	0.788***	0.769	0.852	0.591
	OE3	0.798***					KP2	0.725***			
	OE4	0.802***					KP3	0.761***			
UE	UE1	0.767***	0.786	0.862	0.609		KP4	0.799***			
	UE2	0.793***				PC	PC1	0.737***	0.709	0.821	0.534
	UE3	0.768***					PC2	0.738***			
	UE4	0.794***					PC3	0.744***			
RE	RE1	0.775***	0.791	0.864	0.614		PC4	0.702***			
	RE2	0.801***									
	RE3	0.768***									
	RE4	0.790***									
CO	CO1	0.777***	0.865	0.896	0.552	RS	RS1	0.760***	0.719	0.826	0.542
	CO2	0.758***					RS2	0.732***			
	CO3	0.766***					RS3	0.703***			
	CO4	0.704***					RS4	0.748***			
	CO5	0.736***									
	CO6	0.720***									
	CO7	0.740***									

HOC	Items	Indicator loading	CA	CR	AVE	HOC	Items	Indicator loading	CA	CR	AVE
EI	SE	0.740***	0.764	0.849	0.585	CTD	CO	0.876***	0.608	0.835	0.717
	OE	0.799***					RS	0.817***			
	UE	0.744***									
	RE	0.776***									
TPI	PS	0.828***	0.692	0.829	0.618						
	KP	0.746***									
	PC	0.783***									

***p < 0.001, SE, self-emotion appraisal; OE, others-emotion appraisal; UE, use of emotion; RE, regulation of emotion; PS, professional self-efficacy; KP, knowledge about the profession; PC, professional commitment; CO, critical openness; RS, reflective scepticism; EI, emotional intelligence; TPI, teacher professional identity; CTD, critical thinking disposition.

**Table 4:** Summary of discriminant validity.

LOC	CO	KP	OE	PC	PS	RE	RS	UE	SE
CO	**0.743**	0.412	0.391	0.482	0.452	0.367	0.553	0.383	0.367
KP	0.337	**0.769**	0.349	0.545	0.518	0.283	0.386	0.311	0.306
OE	0.325	0.272	**0.779**	0.369	0.455	0.652	0.408	0.553	0.578
PC	0.380	0.404	0.278	**0.731**	0.601	0.323	0.400	0.338	0.341
PS	0.387	0.418	0.369	0.463	**0.775**	0.423	0.448	0.385	0.376
RE	0.305	0.223	0.514	0.245	0.346	**0.784**	0.350	0.532	0.572
RS	0.437	0.288	0.311	0.286	0.348	0.268	**0.736**	0.305	0.287
UE	0.318	0.242	0.435	0.254	0.315	0.420	0.230	**0.781**	0.577
SE	0.294	0.233	0.439	0.247	0.296	0.437	0.212	0.439	**0.746**
HOC	CTD	EI	TPI						
CTD	**0.847**	0.640	0.781						
EI	0.441	**0.765**	0.632						
TPI	0.512	0.466	**0.786**						

SE, self-emotion appraisal; OE, others-emotion appraisal; UE, use of emotion; RE, regulation of emotion; PS, professional self-efficacy; KP, knowledge about the profession; PC, professional commitment; CO, critical openness; RS, reflective scepticism; EI, emotional intelligence; TPI, teacher professional identity; CTD, critical thinking disposition. The bold diagonal values should be “the square roots of the AVE”.

### 4.3 Test of the PLS-SEM structural model

**Figure 3:**
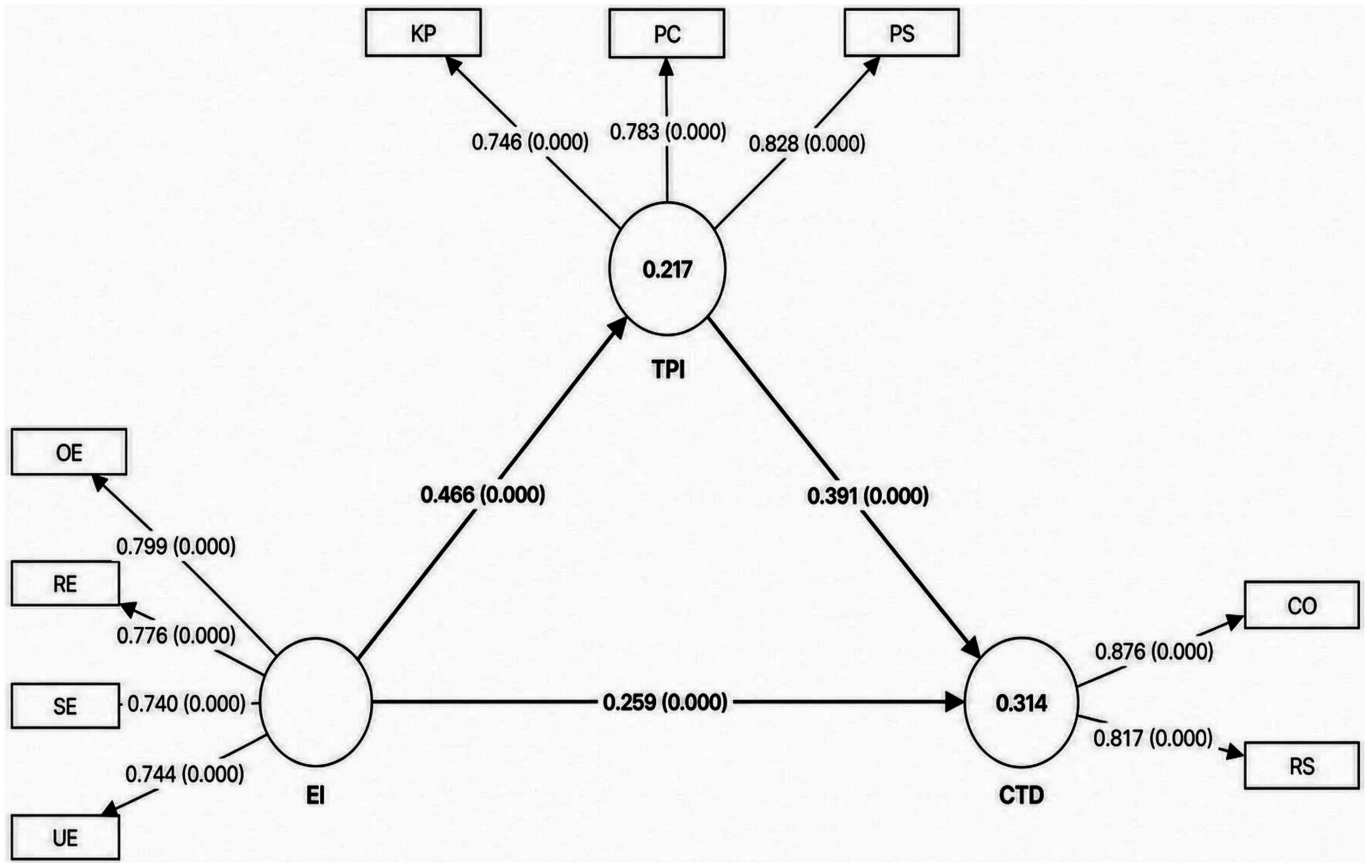
Structural model of EI, TPI, and CTD.

Figure 3 and Table 5 summarise the structural model results, including path coefficients, effect sizes, predictive relevance, and explanatory power. Specifically, EI (*β* = 0.259, *p* < 0.001), together with TPI (*β* = 0.391, *p* < 0.001), showed positive relations to CTD, and EI also acted as a positive predictor for TPI (*β* = 0.466, *p* < 0.001). These results supported the H_1_, H_2_, H_3_, respectively. EI exerted a small effect on CTD (*f*^2^ = 0.076), whereas its effect on TPI was medium to large (*f^2^* = 0.278), while TPI showed a moderate effect on CTD (*f*^2^ = 0.175). All Q^2^ values obtained from cross-validated redundancy were positive, indicating predictive relevance (Hair, 2017). The model explained 21.7% of the variance in TPI (*R*^2^ = 0.217) and 31.4% in CTD (*R*^2^ = 0.314), indicating good explanatory power (Hair et al., 2019). Model fit, assessed via the Standardised Root Mean Square Residual (SRMR; Hair, 2017), was acceptable (SRMR = 0.078 < 0.080), further supporting model adequacy.

**Table 5:** Test of the structural model.

Construct path	Variance inflation factor	Standardised path coefficients	95% confidence interval		*f* ^2^	*R* ^2^	*Q* ^2^	SRMR
			Lower	Upper				
EI - > CTD	1.278	0.259***	0.207	0.312	0.076	0.314	0.222	0.078
TPI - > CTD	1.278	0.391***	0.342	0.439	0.175			
EI - > TPI	1.000	0.466***	0.421	0.509	0.278	0.217	0.131	

***p < 0.001, EI, emotional intelligence; TPI, teacher professional identity; CTD, critical thinking disposition.

### 4.4 Test of the indirect effect of TPI

Table 6 presents the indirect effect of TPI in the association between EI and CTD. Bootstrapping with 5,000 resamples showed that the indirect effect was statistically significant (indirect effect = 0.182, *p* < 0.001), with the 95% confidence interval excluding zero [CI =(0.154, 0.213)]. This result provided statistical support for H_4_. This indirect effect accounted for 41.3% of the overall EI-CTD association. Specifically, the findings indicate that EI may be associated with CTD partly through its association with TPI.

**Table 6:** Test of TPI’s indirect effect.

Indirect path	Indirect estimate	95% confidence interval		Direct estimate	Total estimate	Ratio
		Lower	Upper			
EI - > TPI - > CTD	0.182***	0.154	0.213	0.259***	0.441***	0.413

***p < 0.001, EI, emotional intelligence; TPI, teacher professional identity, CTD, critical thinking disposition, ratio refers to the proportion of the indirect effect relative to the total effect.

### 4.5 Necessary condition analysis

**Figure 4:**
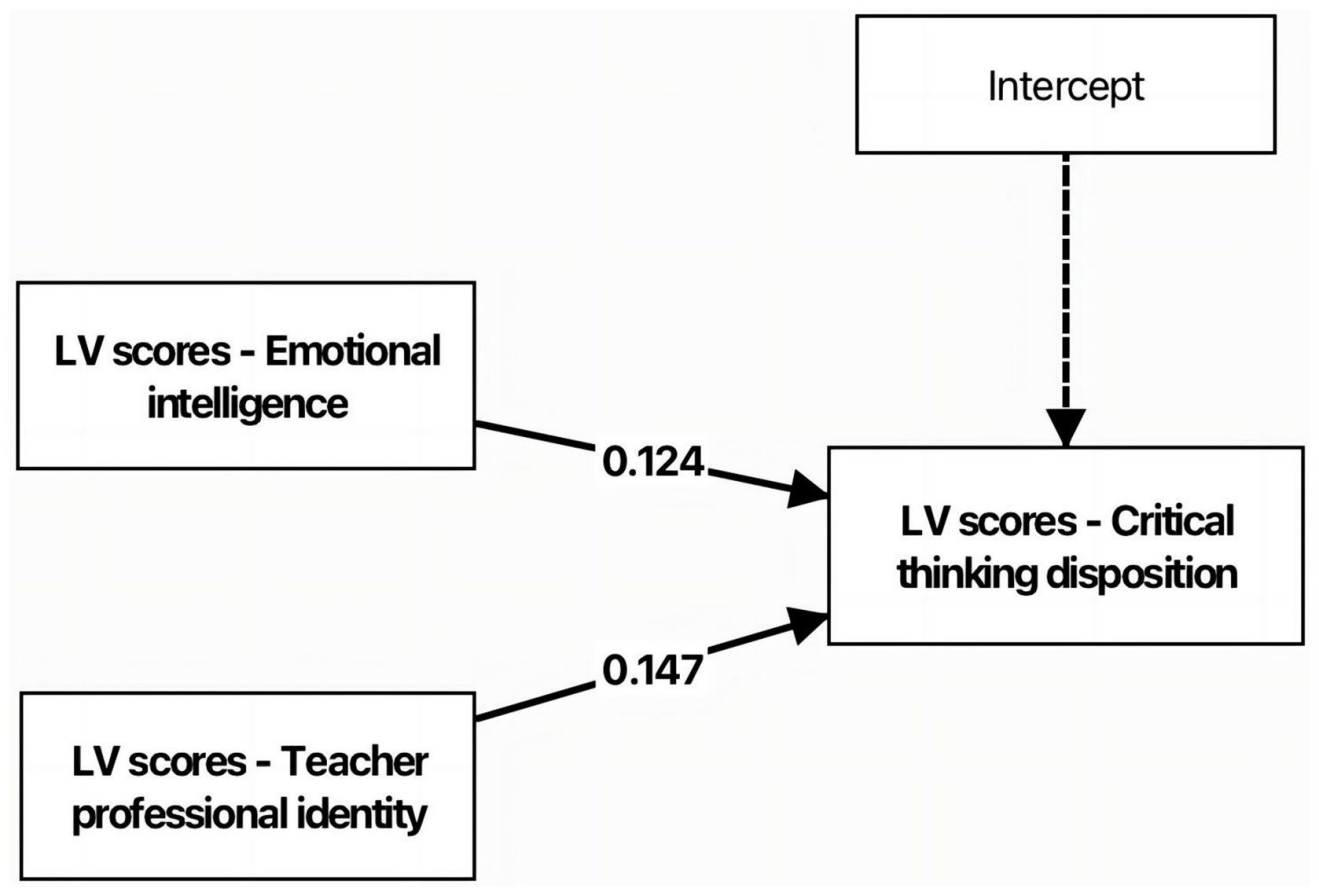
The NCA model.

**Figure 5:**
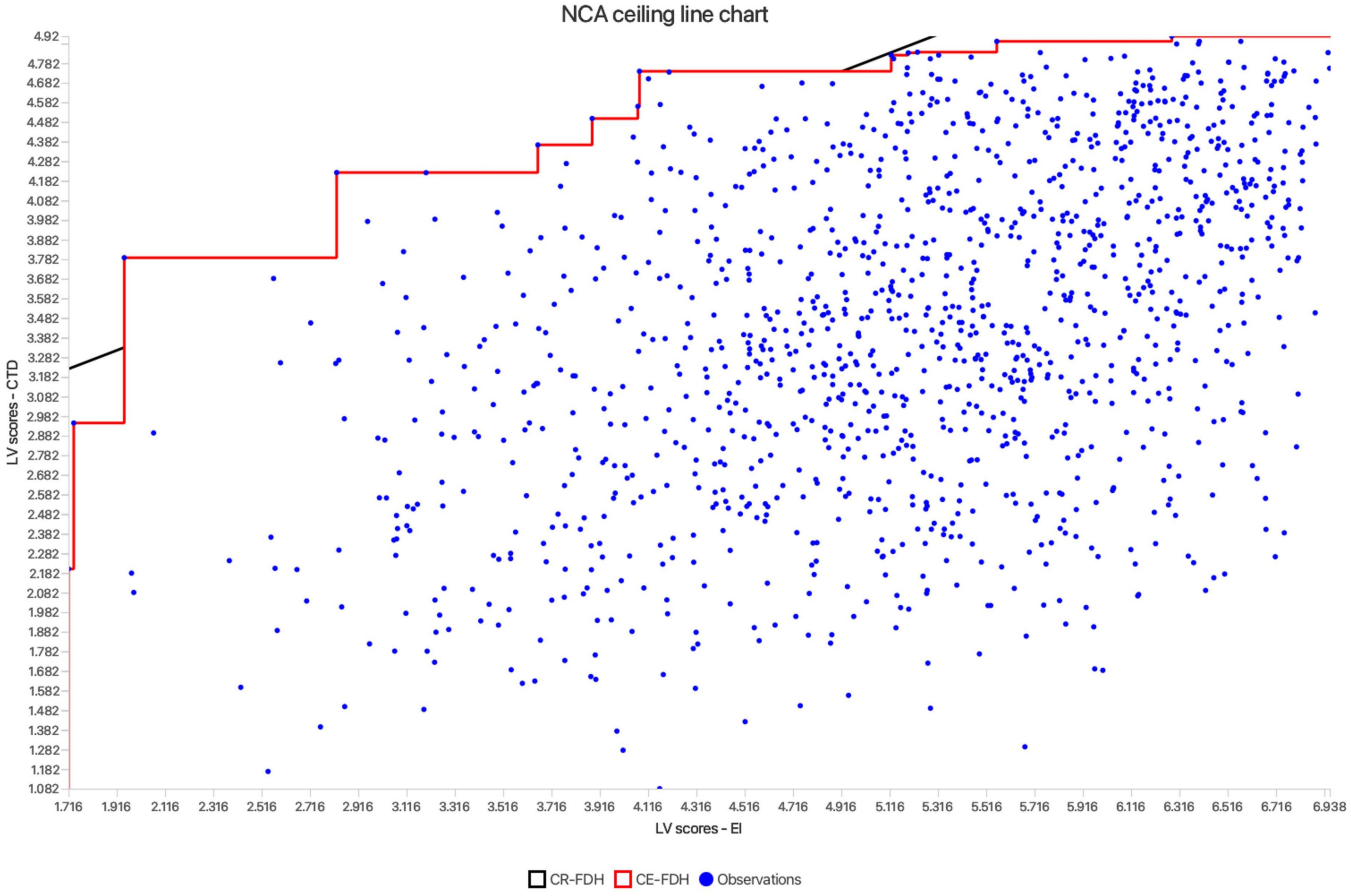
Ceiling line plot (EI-CTD).

**Figure 6:**
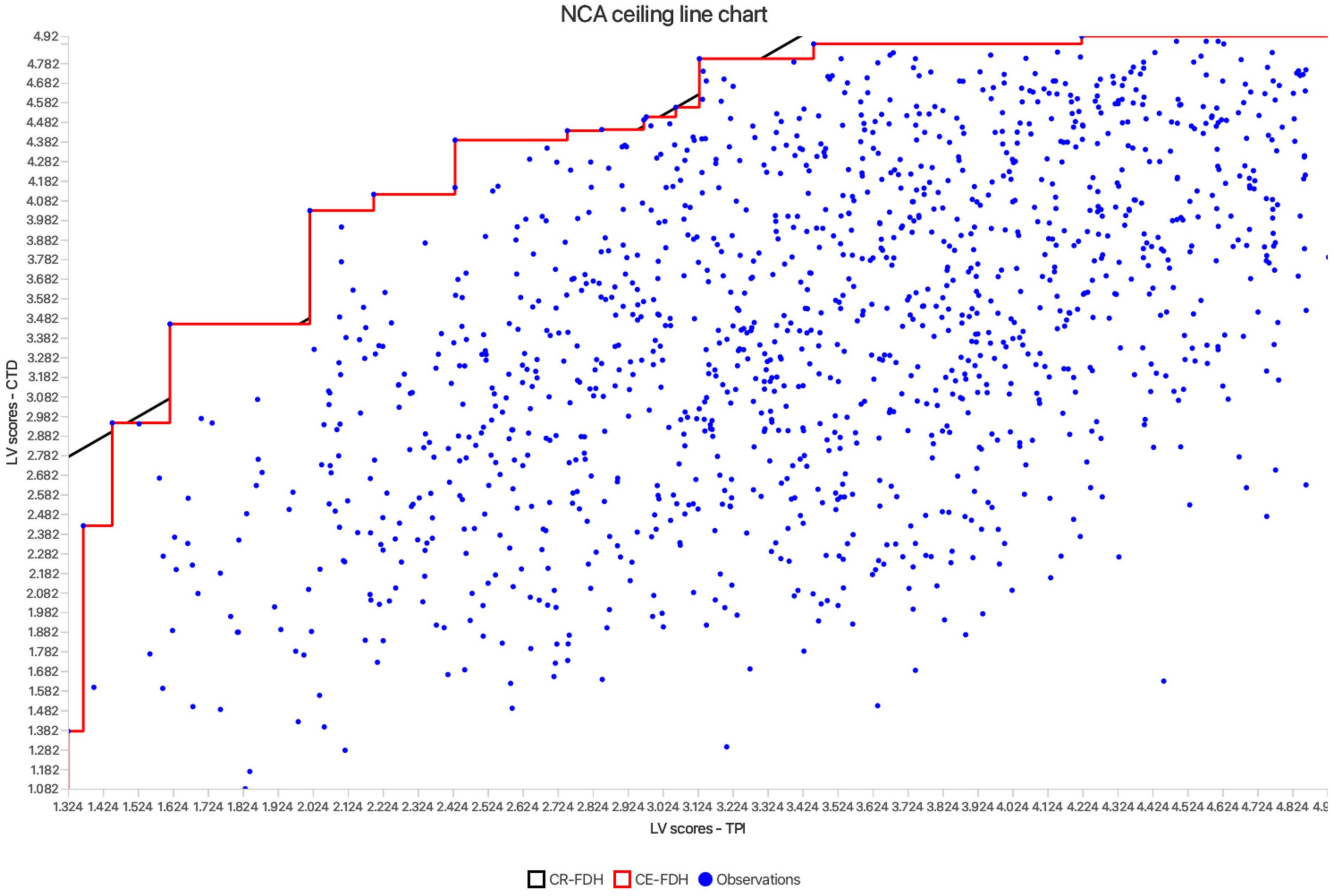
Ceiling line plot (TPI-CTD).

Following the recommended analytical approach by Dul (2016), latent variable scores for EI, TPI, and CTD were further extracted to construct the NCA model (see Figure 4). Subsequently, the NCA procedure in SmartPLS 4.0 was executed, and visual representations in the form of scatterplots were created. As presented in Figures 5, 6, noticeable empty zones appear within the upper-left areas of the scatter plots, indicating regions where no observed data points are present. The unobserved region shows that when EI and TPI remain at low values, high CTD are not observed, with higher CTD values only emerging once these variables exceed a certain threshold. Thus, the results imply that EI and TPI might constitute necessary conditions for pre-service teachers’ CTD within the current sample.

To evaluate the magnitude and significance of the necessity effects, 10,000 permutation-based resampling iterations were performed under the CE-FDH criterion. Therefore, the accuracy was automatically set to 100% (Dul et al., 2020). As shown in Table 7, EI exhibited a statistically significant, medium-level necessity effect on pre-service teachers’ CTD (*d* = 0.124, *p* < 0.001), while TPI also displayed a similarly moderate and significant necessity effect (*d* = 0.147, *p* < 0.001). These findings further supported that EI and TPI each served as necessary conditions for CTD within the current sample.

**Table 7:** Assessment of the necessity effects of EI and TPI.

Constructs	Critical thinking disposition (CTD)		
	Accuracy (%)	Necessity effect size (d)	*p*-value
Emotional intelligence (EI)	100	0.124	0.000
Teacher professional identity (TPI)	100	0.147	0.000

The bottleneck table identifies the minimum levels each condition variable may need to be attained to enable the outcome variable to reach specified thresholds. This delineates the extent to which conditions impose boundary constraints on the outcome within the current sample (Dul, 2016). In Table 8, all values are model-estimated weighted latent variable scores rather than raw item scores. These scores follow the same direction and approximate scale range as the original response categories, with EI measured on a 1–7 scale and TPI and CTD measured on 1–5 scales. Thus, the minimum EI and TPI values listed in Table 8 can be interpreted as approximate construct-level thresholds on their respective original scale ranges. For instance, to achieve a high CTD level (80%; Richter et al., 2023), EI would need to reach at least 2.827 on the 1–7 EI scale, while TPI would need to reach at least 2.431 on the 1–5 TPI scale. Within the present sample, attaining the highest CTD level was associated with minimum EI and TPI values of 6.285 and 4.222, respectively. Values below these thresholds would make the corresponding CTD level unlikely.

**Table 8:** Bottleneck table for NCA results.

Target CTD level (%)	CTD	EI	TPI
0	1.082	NN	NN
10	1.466	NN	1.368
20	1.850	NN	1.368
30	2.234	1.738	1.368
40	2.617	1.738	1.451
50	3.001	1.947	1.615
60	3.385	1.947	1.615
70	3.769	1.947	2.015
80	4.152	2.827	2.431
90	4.536	4.074	3.061
100	4.920	6.285	4.222

NN, Not necessary; EI, emotional intelligence; TPI, teacher professional identity; CTD, critical thinking disposition.

## 5 Discussion

### 5.1 Analysis of the findings

Based on a sample of Chinese pre-service teachers, this study integrated PLS-SEM and NCA to examine the associations among EI, TPI, and CTD from both predictive and necessity-based perspectives. The PLS-SEM results showed that EI and TPI were positively associated with CTD, and that the indirect effect through TPI was statistically significant. These findings suggest that emotional resources and professional identity may be relevant to pre-service teachers’ dispositional willingness to engage in reflective, open-minded, and evidence-oriented thinking. In addition, the NCA results indicated that low levels of EI and TPI may constrain the attainment of higher CTD within the present sample. Thus, rather than duplicating the PLS-SEM findings, NCA provided a complementary perspective by identifying potential boundary conditions under which high CTD scores were unlikely to occur. Together, these findings offer a more nuanced understanding of how EI and TPI are related to CTD in pre-service teachers.

From the predictive perspective, the present study found that EI was positively associated with CTD among pre-service teachers. This observation is consistent with previous studies conducted with other populations (Kang, 2015; Sk and Halder, 2020; Zhao et al., 2025a; Zhao and Ai, 2026). A more coherent explanation can be developed by treating Salovey and Mayer’s (1990) EI model as the primary theoretical lens, while using social-emotional learning and Broaden-and-Build perspectives as complementary accounts of how emotional competence supports reflective cognition. According to Salovey and Mayer’s (1990) model, EI involves perceiving, understanding, using, and regulating emotions. These capacities may help pre-service teachers recognise affective cues, regulate anxiety or frustration, and use emotional information constructively when facing pedagogical uncertainty, interpersonal tension, or competing viewpoints (Mérida-López and Extremera, 2017). From a social-emotional learning perspective, such emotional competencies are closely connected with responsible cognitive engagement (Hoffman, 2009). The Broaden-and-Build view further clarifies why emotionally regulated or positive affective states may expand cognitive flexibility and openness to alternative possibilities (Fredrickson, 2001). Taken together, these perspectives suggest a focused explanatory pathway: EI may support more stable emotional regulation and constructive use of affective information, which in turn helps pre-service teachers remain open, reflective, and evidence-sensitive under uncertain teaching-related conditions. These qualities are central to CTD, which involves openness to alternative perspectives, reflective scepticism, and willingness to revise initial judgements in light of evidence (Facione, 1990; Zhao et al., 2024).

The present study also found that TPI mediated the association between EI and CTD among pre-service teachers. This finding not only extends the discussion of the linkage between affective and cognitive dispositions, but also suggests that pre-service teachers’ higher-order cognitive dispositions may be partly understood through value-based identity construction. Given the cross-sectional design of the study, this mediating effect should be interpreted as an indirect statistical association rather than as evidence of temporal or causal ordering. The present finding is consistent with prior literature suggesting that teachers with higher EI are generally more capable of perceiving and regulating their own emotions (Su et al., 2024; Yin et al., 2013; Yin, 2015). From a self-efficacy perspective, emotional states may serve as important cues for self-efficacy judgements (Bandura, 1977). Pre-service teachers with higher EI may be more capable of emotional regulation and emotional understanding, which may help them accumulate more positive experiences in teaching-related contexts and strengthen their belief in their professional competence (Long et al., 2026). Professional self-efficacy is also a core component of TPI (Zeng and Liu, 2024), which may help explain the association between EI and TPI.

It is also noteworthy that CTD involves the willingness to engage in complex information processing, evaluative reflection, and reasoned judgement, which may require a certain degree of cognitive confidence (Facione, 1990; Zhao et al., 2024). Liu and Pásztor (2022) similarly identified self-efficacy as an essential component of CTD. When pre-service teachers experience greater efficacy in their professional learning and teaching-related activities, they may be more likely to maintain cognitive confidence and internalise professional values aligned with their teacher identity, such as truth-seeking, rationality, reflection, and critical awareness. These value-based orientations may, in turn, be associated with stronger CTD. Moreover, prior research has shown that teachers with a stronger sense of professional identity tend to employ more diverse problem-solving strategies and exhibit greater creativity and cognitive flexibility when faced with complex issues (Ding et al., 2022; Su et al., 2024), which may further support their reflective and critical orientations. This finding underscores the importance of not only cultivating emotional competencies for pre-service teachers but also concurrently fostering their robust sense of professional identity. Such integration of emotional, value-based, and cognitive dimensions may better support the strengthening of CTD among pre-service teachers.

As a key methodological contribution, this study introduced NCA to complement the explanatory scope of traditional path analysis. Beyond examining average predictive associations, the NCA results suggested that EI and TPI may operate as necessary conditions for attaining higher CTD scores within the present sample. This offers additional empirical evidence that EI and TPI are not only positively associated with CTD, but may also represent potential limiting conditions for higher levels of CTD among pre-service teachers. Specifically, when pre-service teachers’ EI or TPI falls below certain threshold levels, higher CTD scores appear less likely to occur. This finding is consistent with the central logic of NCA, which focuses on whether the absence or insufficiency of a condition constrains the upper limit of an outcome (Richter et al., 2023).

Theoretically, the necessity result for EI further suggests that emotional competencies may provide an important basis for maintaining openness, reflective engagement, and reasoned judgement (Facione, 1990; Sk and Halder, 2020). Similarly, the necessity result for TPI highlights the potential importance of professional self-concept and value alignment in relation to pre-service teachers’ CTD. From an identity-based perspective, the salience of professional identity may set important boundaries for the extent to which pre-service teachers align their cognitive orientations and behavioural tendencies with the reflective and evidence-informed expectations of the teaching profession (Bowskill, 2017). This may partly explain why low levels of TPI appear to constrain the attainment of higher CTD scores in the present sample. Practically, the bottleneck analysis specifies the minimum levels of EI and TPI associated with different CTD thresholds, suggesting that pre-service teachers with relatively low emotional or identity-related resources may require additional support when seeking to foster higher CTD. However, although the necessity effects of EI (*d* = 0.124) and TPI (*d* = 0.147) reached the medium range according to NCA conventions, they still represent modest constraints in substantive terms. The identified thresholds should therefore be interpreted as exploratory, sample-specific boundary estimates rather than strong diagnostic cut-off points, and their practical significance should be considered cautiously across different populations and educational contexts.

Importantly, the purpose of NCA is not to show that increasing a condition will automatically enhance the outcome, nor does it establish causal effects. Rather, it identifies whether insufficient levels of a condition may restrict the possibility of achieving higher outcome levels. Therefore, interpreting EI and TPI as necessary conditions for CTD does not mean that improving EI or TPI alone will necessarily lead to higher CTD. Instead, the findings suggest that, within this dataset, low levels of EI and TPI may function as potential constraints on the attainment of higher CTD scores.

### 5.2 Educational implications

At the theoretical level, this study contributes to the literature by broadening the range of factors considered in relation to CTD among pre-service teachers. Previous research has often examined CTD in connection with cognitive characteristics, learning experiences, or pedagogical interventions, whereas the present study brings EI and TPI into the discussion. This perspective is particularly relevant for pre-service teachers, who are still developing their emotional understanding, professional role perceptions, and cognitive orientations towards educational problems. By linking EI and TPI with CTD, the study helps frame CTD not merely as an isolated cognitive disposition, but as a construct that may also be related to how future teachers regulate emotions, interpret the meaning of the teaching role, and attach personal value to professional judgement. In doing so, the study incorporates affective and identity-related dimensions that have received comparatively limited attention in CTD research, thereby contributing to a more integrated theoretical account of CTD in pre-service teachers.

At the practical level, the findings suggest several possible directions for supporting CTD among pre-service teachers. In relation to the association between EI and CTD, emotion-related learning activities could be designed with reference to established social–emotional learning and teacher emotion-regulation programmes. For example, the RULER approach provides a structured framework for recognising, understanding, labelling, expressing, and regulating emotions (Brackett et al., 2012; Rivers et al., 2013), while the CARE for teachers programme offers mindfulness and emotion-regulation-based practices for strengthening teachers’ social and emotional competence (Jennings et al., 2017). Adapted to pre-service teacher context, such models could inform scenario-based discussions, video analysis, reflective writing, and emotional regulation exercises in classroom-management or practicum-related modules.

The findings also point to the potential value of supporting TPI cultivation through structured identity-development models rather than relying only on informal reflection. For example, Korthagen and Vasalos’s (2005) core reflection model encourages teachers to connect concrete teaching experiences with deeper layers of professional functioning, including beliefs, identity, and mission. In pre-service teacher programmes, this model could guide reflective discussions on practicum experiences, career motivation interviews, mentoring conversations, and identity-related writing tasks. Such activities may help pre-service teachers connect their learning experiences with their emerging sense of professional responsibility, thereby strengthening the value-based basis for CTD.

The NCA findings offer further practical insight, although they should be interpreted cautiously because the observed necessity effects represent modest constraints rather than strong limiting conditions. Teacher educators may therefore use bottleneck thresholds identified in their own survey samples only as exploratory reference benchmarks, not as fixed screening criteria. Such information may help inform more responsive support for students whose emotional regulation or professional identity development may require further attention.

### 5.3 Limitations

Several limitations should be acknowledged in this study. Although the use of a cross-sectional design was useful for identifying significant associations among EI, TPI, and CTD, it does not allow robust causal inference or establish the temporal ordering of these constructs. Therefore, the mediating role of TPI should be interpreted as an indirect statistical association rather than evidence of causal mediation, and future research may adopt longitudinal or experimental approaches to more rigorously examine the directionality of these relationships. Moreover, reliance on self-reported questionnaire data may give rise to social desirability effects, biases in self-perception, and possible common method variance, while such measures are commonly used to assess psychological constructs. Although the single-factor test suggested that common method variance was unlikely to dominate the observed relationships, this diagnostic approach has inherent limitations. Future studies could address this concern more directly by incorporating an *a priori* marker variable, comparing models with and without a latent method factor, or using behavioural indicators, scenario-based tasks, evaluations from teacher educators, multi-source data, or time-lagged designs to provide more objective and triangulated evidence. Another limitation relates to the transferability of the NCA results. Because the results largely depend on the present sample, measurement instruments, and data boundaries, their applicability beyond this study remains uncertain. This limitation is also related to the sampling scope, as participants were recruited from three universities in south-western China. In this context, Confucian-influenced educational values, such as respect for teachers, moral cultivation, effort, and role responsibility, may influence how pre-service teachers understand professional identity and thinking disposition. Institutional expectations around becoming a qualified teacher may also have encouraged alignment with collective professional norms, which could influence the observed relationships among EI, TPI, and CTD. Future studies involving larger and more heterogeneous samples across different institutions and sociocultural settings are therefore needed to assess the robustness of the identified associations and necessity patterns.

## 6 Conclusion

This study examined the relationships among emotional intelligence (EI), teacher professional identity (TPI), and critical thinking disposition (CTD) among Chinese pre-service teachers, while also exploring whether EI and TPI may function as necessary conditions for higher CTD scores. The findings show that pre-service teachers’ EI, TPI, and CTD are positively related, and that TPI serves as an indirect statistical link between EI and CTD. The NCA results further suggest that insufficient levels of EI and TPI may constrain the attainment of higher CTD within the present sample. Taken together, the predictive and necessity-based findings suggest that CTD cultivation among pre-service teachers may involve both additive psychological resources and minimum enabling conditions that make sustained reflective, open-minded, and evidence-sensitive thinking more attainable. These findings highlight emotional competence and professional identity might as interconnected psychological foundations for CTD. Future research should further examine, preferably through longitudinal or intervention designs, whether strengthening EI and TPI can create more favourable conditions for CTD development across diverse pre-service teacher populations.

## Data Availability

The raw data supporting the conclusions of this article will be made available by the authors, without undue reservation.
